# What Works, for Whom, in What Circumstances and Why, When Integrating Voluntary and Statutory Community Mental Health Services: A Realist Evaluation Case Study

**DOI:** 10.5334/ijic.9060

**Published:** 2025-09-30

**Authors:** Charley Hobson-Merrett, Rebecca Hardwick, Jane Yeandle, Beccy Wardle, Catherine Connor, John Gibson, Vanessa Pinfold, Alex Stirzaker, Richard Byng

**Affiliations:** 1 University of Plymouth, UK; 2 Somerset NHS Foundation Trust, UK; 3 Rethink Mental Illness, UK; 4 Somerset Integrated Care Board, UK; 5 The McPin Foundation, UK

**Keywords:** integrated care, open mental health, mental health, realist evaluation, lived experience, voluntary sector

## Abstract

**Background:**

Integration of voluntary and statutory mental health services may address gaps in mental health care. The Community Mental Health Framework for Adults and Older Adults 2019 provided impetus for integration within England. It was unknown how, why, or under what circumstances integration would occur.

**Methods:**

A realist evaluation of Framework implementation in Somerset was undertaken. The extent of change, and how, why and under what circumstances change occurred were evaluated. Embedded researchers collected observational and interview data, and reviewed internal records. Realist qualitative analysis created and tested a programme theory exploring the extent, cause, and impact of change.

**Results:**

Services worked together to create an integrated mental health service using a cultural change model: new language prompted new ways of thinking and working. Programme theory testing demonstrated good extent of change. Voluntary sector integration helped address treatment gaps: all service users were offered a service. Mechanisms of change included: new language, relationship building, flexible working, and valuing voluntary sector services. Contextual factors affecting the extent of change included: balances of power, rigidity of statutory services, and trusting management.

**Conclusions:**

Integrating services using a culture change model addresses gaps in mental health care. Optimal implementation requires addressing contextual barriers.

## 1. Introduction

### 1.1 Background

Internationally, prevalence of mental health (mh) problems is growing [1,2]. There are substantial treatment gaps with many left waiting or without support [3]. Delays in MH care treatment are linked to increased symptoms (physical health, depression, psychosis), increased incidences of harmful behaviour (aggression, self-harm, substance misuse), and reduced socialisation [4,5]. Denials of care and fractured care pathways reduce patient satisfaction [6]. In England, denials of care by one service, may lead to referral to another service, and constant reassessment often not resulting in the provision of support. Reassessment can have negative impacts on service users (SUs), including feelings of frustration and being punished, or that the clinician is trying to ‘catch them out’ [7]. International shifts towards biopsychosocial understandings of MH problems has broadened definitions of MH ‘treatment’ [8,9,10]. For example, tackling psychosocial causes of poor MH such as housing, finances, ability to form and maintain relationships, loneliness, gaining and retaining meaningful employment. Often these non-medical psychosocial interventions are provided by the voluntary sector (VCSEs). This creates an opportunity for medical and VCSE services to work together to close treatment gaps by providing interventions that contribute to tackling these psychosocial causes of mental health problems. Furthermore, integrated working within statutory medical services, also provides an opportunity to improve the quality of services offered, for example, reducing reassessment burden.

Within England, changes to national policy, including the NHS Long Term Plan 2019 and the Community Mental Health Framework for Adults and Older Adults 2019, provided an impetus for statutory and VCSEs to work together to address gaps in MH care [11,12]. These policies anticipate that integration between and within statutory (primary and secondary care) and VCSEs will contribute to improved support for people with MH problems. However, policies did not give clear guidelines regarding implementation of integration, or evidence regarding how integration will impact service provision. This paper evaluates the use of a cultural change approach to implementing the Community Mental Health Framework, via statutory-VCSE integration.

### 1.2 Problem Statement

It is unclear how to effectively implement statutory-VCSE integration, or how integration impacts service provision.

Ethical Approval: Approved by Somerset NHS Foundation Trust Research & Development.

## 2. Description of the Practice

Somerset is a county in England (population approximately 571,000, predominately rural). Somerset developed and implemented the Open Mental Health (OMH) model to address the need for integrated working.

### 2.1 Development of OMH

Experts by Experience (EbEs) and representatives from primary care, secondary care, social care, commissioners, and VCSEs worked together to form the Open Mental Health Alliance (the ‘Alliance’) [13]. The Alliance is led by a steering group of representatives from each organisation (Somerset NHS Foundation Trust, Somerset Integrated Care Board (ICB), Somerset Council, Rethink Mental Illness, and Primary Care Networks), and EbEs. Together, the Alliance provides Open Mental Health (OMH): an integrated service that provides people with MH problems with “the right care at the right time”.

To support the provision of the right care at the right time, a needs-based assessment tool (DIALOG+) was adopted across OMH [14]. In DIALOG+ people rate their satisfaction across seven domains; low stratification prompts a conversation between the staff member and SU regarding appropriate ways to address these needs. This includes drawing on the SU’s own resources; as such DIALOG+ also acts as a needs-based intervention. DIALOG+ was intended to replace other assessment tools across the OMH, moving staff from assessing whether their service is the appropriate support to thinking about how OMH as a whole can provide appropriate support.

A sub-alliance of VCSE organisations (the ‘VCSE Alliance’), was formed. At the time of writing the VCSE Alliance included: Age UK Somerset, The Balsam Centre, Citizens Advice Somerset, Rethink Mental Illness, Mind in Somerset, Second Step, Watch CIC, Somerset and Wessex Eating Disorder Association (SWEDA), Spark Somerset, Young Somerset, Diversity Voice, 2BU, Minehead Eye, Conquest Centre, Fuse Performance, and Somerset Activity and Sports Partnership. Appendix 1 outlines the services provided by the VCSE Alliance. To address VCSE/statutory sector power imbalances and to streamline commissioning, the VCSE Alliance is commissioned via a subcontracting arrangement between Rethink Mental Illness and Somerset ICB. To address power imbalances between different size VCSEs there is an agreement to favour local charities over national charities in funding allocations.

Somerset secondary care provision is organised into four geographical ‘localities’. OMH is organised around these localities, creating place-based services: new ‘Primary Care Liaison’ staff were employed to reduce barriers between Secondary and Primary Care in each locality; a lead VCSE organisation in each locality aided local integration between Secondary Care and local VCSEs; existing Statutory Community Mental Health Services began to work closely with local VCSE Alliance organisations. At county-level integration included representation and contribution to strategic OMH meetings, and collaboration over ongoing funding. At locality integration included working together to decide the most appropriate service(s) to offer an individual, and if appropriate, working together to provide a complementary combination of services to individuals. At locality level integration also included some incidences of cross-organisational supervision. Post COVID-19 co-location of some secondary care services was implemented to enhance joint working; co-location across primary/secondary care and secondary care/VCSEs was not enacted within the time scale of the evaluation.

### 2.2 Implementation of OMH

Ensuring strategic and commissioning changes translated into changes in service provision required staff at all levels to embrace the benefits and ethos of integration, and make changes to their working practices. Somerset adopted a ‘cultural change’ approach to implementing change: addressing how staff work, think, and behave [15].

**Figure 1:**
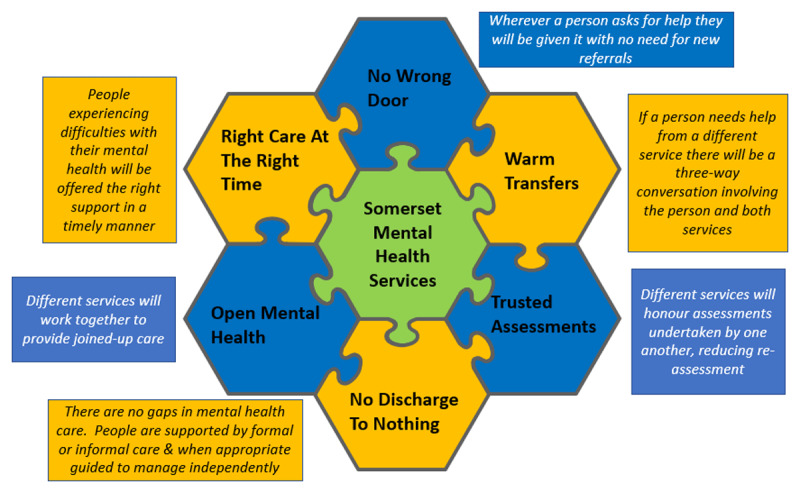
Expected Cultural Changes.

**Figure 2:**
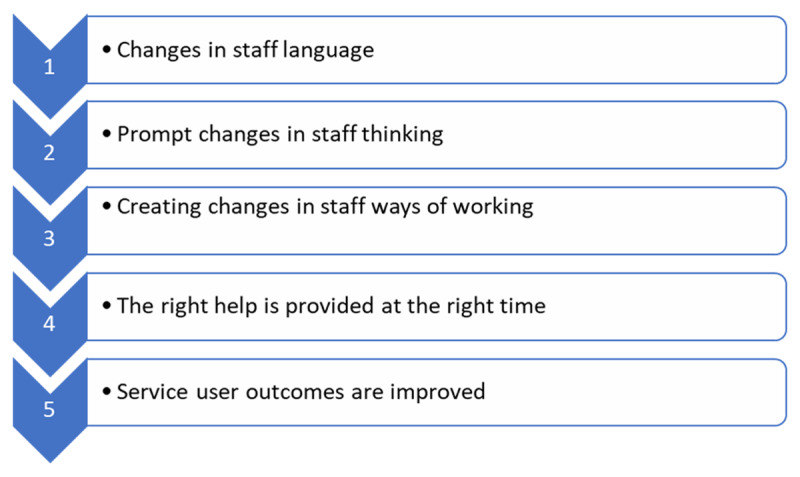
High level initial programme theory.

A strategic level top-down approach to cultural-change was designed by the OMH Steering Group. This involved agreeing, defining, and adopting a set of new expected behaviours, underpinned by new language, to create change in the ways staff in the ecosystem provide services. Researchers undertook stakeholder engagement, via informal conversations and observations with programme architects, to create an understanding of the expected cultural changes (Figure 1) and how these changes were expected to take place (Figure 2). Briefly, new language was expected to prompt staff to think and work in the new, integrated, ways, and these new ways of working would lead to improvements in service provision. This integration meets a whole-system, a decision-maker, and a process-based definition of integrated care [16,17,18,19].

## 3. Evaluation methods

### 3.1 Setting: Mendip

Evidence suggests that clinical micro-systems create contextual differences in successful implementation of health care quality improvements [18]. Therefore, to explore the implementation of integration in contextual detail, one locality was selected as a site: Mendip. Mendip covers four primary care networks, approx. population 119,000. Mendip’s VCSE partners are: Second Step, Age UK Somerset, Citizens Advice Somerset, Mind in Somerset, SWEDA, and Watch CIC.

### 3.2 Researcher-in-residence

A researcher-in-residence (RiR) approach [19] was used. Researcher-in-residence models capitalise on both the insider knowledge of in-house evaluations and university academic expertise. A University of Plymouth researcher (CHM) led the evaluation, working with a McPin Foundation peer researcher (JG), and an assistant psychologist from Somerset NHS Foundation Trust (LR). The RiR team become honorary members of OMH staff, building strong relationships with the OMH Steering Group, Mendip OMH staff and managers. Honorary contracts allowed RiRs to acess statutory service data through normal governance procedures. This evaluation was co-designed by the RiRs and OMH Steering Group. RiR supervision was provided by University of Plymouth (RH, RB), the McPin Foundation (VP) and NHS England (AS). Due to COVID-19 restrictions, RiRs were embedded remotely, reflecting the teams they were embedded in.

### 3.3 Realist evaluation

Realist evaluation [20] is a theory-driven approach which acknowledges that change is generated by hidden ‘mechanisms’ (e.g. powers, forces, liabilities), and these mechanisms are affected by context, which enable or disable changes [21,22,23]. Different things work for different people, in different places, and at different times: changes will work better or be more complete under some circumstances than others. Realists aim to understand what works, under what circumstances, for who it works, and why. This is helpful in adapting practices to different places and people, and understanding how to optimise new practices. This approach also enabled narrowing the focus of the evaluation to one locality, whilst still creating transferable findings with national and international relevance.

### 3.4 Data Collection

Data collection took place between October 2021 and May 2022. CHM led data collection, supported by JG and LR. Data consisted of realist semi-structured interviews, observations of staff meetings, and secondary care patient records. Staff contributed additional data by email and invited RiRs to attend implementation activities.

#### 3.4.1 Interviews

Interviews were undertaken with secondary care and voluntary sector staff. A sample frame of staff within the locality was created. Purposive sampling was undertaken based on testing and refining our initial programme theory (IPT), including: a range of new and established staff; staff employed by both the Trust and VCSE providers; staff in both established roles and new roles. Purposive sampling was intended to explore contextual factors around ‘for whom’ change had occurred. Potential interviewees were contacted by email. To avoid staff feeling pressured into taking part, if a potential interviewee did not reply within two contacts this was understood to mean the person did not wish to be interviewed. At the beginning of each interview the RiR explained: the evaluation; that interviews were confidential and anonymous; that taking part was voluntary; that interviewees could withdraw up to two weeks after interview. RiRs asked interviewees if they had any questions and whether they consented to take part. Consent conversations were recorded and auto-transcribed alongside interviews.

To maximise anonymity in this small sample, the sample frame and the job roles of those interviewed are not reported here. Additionally, quotes included in this report have not been allocated identifiers or pseudonyms, instead being anonymous. Where possible the RiRs selected supporting quotes that did not contain identifiable information. Where quotes containing possibly identifiable information were helpful in aiding understanding, RiRs sought permission from interviewees to include these quotes. Where permission was withheld interviewees could either veto the use of the quote, or supply a re-phrased quote.

CHM designed the interview schedules. Based on the IPT, and with the objective of testing and refining this theory, interview schedules aimed to capture individuals’ contextual factors that may impact cultural change, and the individuals’ experience of the extent of cultural change, including how and why change had taken place. Aligning with realist interviewing [24], the interviewer treated the interviewee as the expert teacher and asked them to explain what was happening in the local system, or asked the interviewee their opinion on early theories regarding how implementation worked. Interviews were undertaken using Microsoft Teams, and were recorded and auto-transcribed using the built-in software in this platform. Professional transcribers checked and corrected these transcripts.

#### 3.4.2 Meeting observations

In Mendip a range of regular, online, meetings took place between representatives of different services. Meetings were sampled based how regularly they occurred; frequent meetings were observed more often.

Meeting attendance required permission and invitation from the chair. Usual invitees were informed of the RiRs’ attendance in advance, and the RiRs and their role was introduced at the beginning of each new meeting they attended.

Although meetings took place online, researchers felt it would be less intrusive and allow observation of natural, usual, ways of working if these meetings were not recorded. To balance rigour with anonymity, and the need to ensure meeting participants felt comfortable to continue with their usual work, RiRs made hand-written notes during meeting observations. These notes were later transferred onto a template based on the six key cultural change elements (Figure 1) and the evaluation objectives. Meeting observations were a data source in their own right, as well contributing to the SU case studies.

#### 3.4.3 SU case studies

Researchers-in-residence used observations from meetings, alongside conversation with statutory staff, to select four SUs for detailed case studies. Service users were sampled purposively from those discussed in observed meetings; aiming to explore knowledge gaps identified in the analysis of meeting and interview data. These case studies used data from meeting observations, and a detailed review of the SUs’ secondary care records to understand what was offered to SUs in the new integrated system, how this compared to what they were offered previously, whether this demonstrated expected cultural changes, and what changes meant for SUs.

Case studies outlined the background of the SU, an overview of any Somerset NHS Foundation Trust mental health services they had been offered prior to implementation of integrated working, and the person’s current needs. The case studies then went on to describe the MH services that the person had been offered post-implementation of integrated working, to what extent this aligned with the six key expected cultural changes, and any differences compared to services offered pre-implementation. Data from these case studies was used as part of the analysis. Whilst extracts from these case studies are included as illustrative points in the findings of this paper, to preserve SU anonymity within a small geographic area, the details of case studies (i.e., a vignette of gender, past and current needs, any diagnoses, previous service use, exact offering provided) are not reported.

LR attempted to contact SUs from these case studies to invite them to interview,. This was unsuccessful: some did not respond to attempts to contact; others responded that their symptoms/needs were currently too severe for them to be interested in taking part. Staff speculated that those who did not respond no longer required MH support, for example, having returned to work and being unavailable to answer phone calls during office hours.

### 3.5 Data analysis

A framework for analysis was created, based on the six key cultural changes. Each piece of data was analysed deductively against this framework, looking for evidence of the extent of cultural change. Data was analysed inductively and retroductively [25] for evidence of mechanisms used to create cultural change, contextual factors that enabled or disabled mechanisms, and the impact of change on SUs. Within this process mechanisms were assumed to consist of a combination of the provision of resources, and people’s responses to these resources (‘reasoning’) [21]. Researchers then looked across the dataset to identify patterns (demi-regularities) that showed the overall extent of cultural change, the mechanisms by which change had happened, any contextual factors that enabled or disabled mechanisms, and the impact of that change on SUs. These were expressed as context-mechanism-outcome configurations (CMOCs), expressing causation by showing how mechanisms led to change, and under what circumstances these mechanisms worked, via a series of if-then statements. These CMOCs were used to refine the IPT.

### 3.6 Patient and public involvement

Patient and public involvement occurred at multiple levels. The service redesign was made in consultation with EbEs employed within Somerset OMH. Experts by Experience took part in initial stakeholder meetings, contributing to the design of this study, and its focus on cultural change and impact on SUs. Alongside the rest of the OMH Steering Group EbEs worked with the RiR to disseminate findings from the evaluation. A peer researcher (JG), employed by the McPin Foundation, worked as an RiR collecting and analysing data, and contributing to the write-up of findings. Additionally, some academically qualified researchers brought their own disclosed and undisclosed lived experience [26].

## 4. Findings

**Figure 3:**
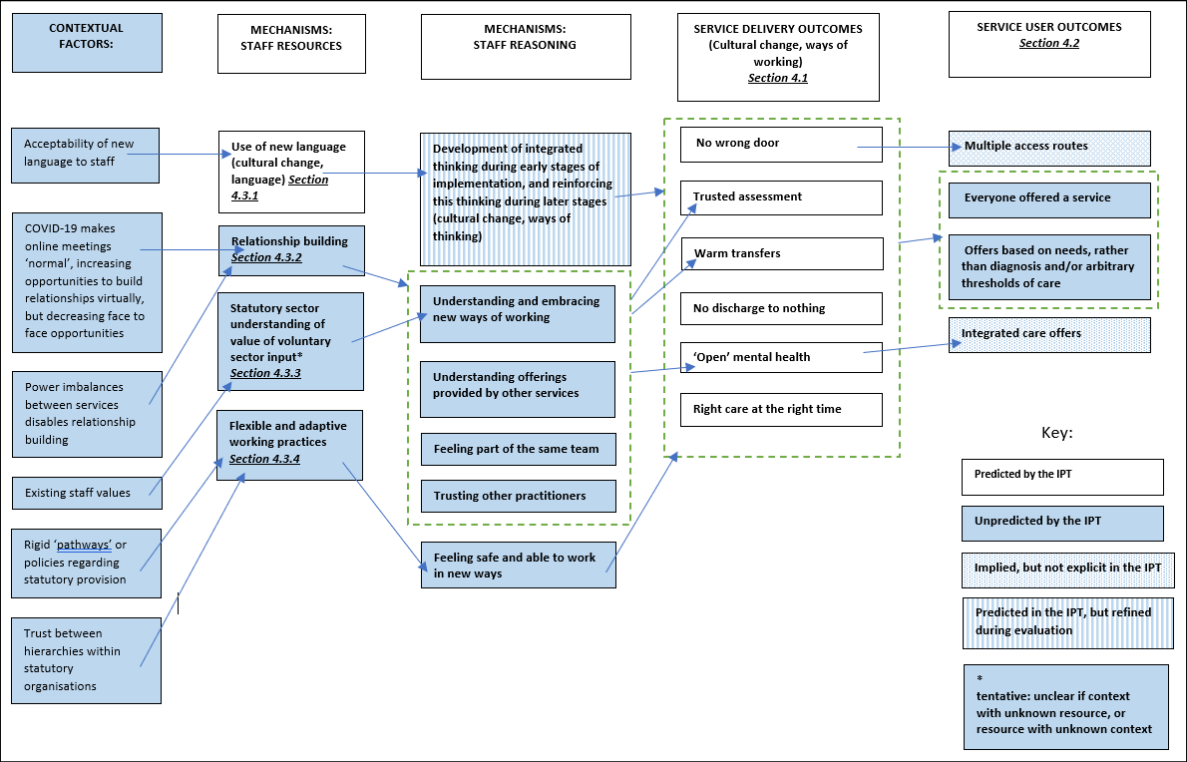
Refined Programme Theory.

Fifteen staff members were interviewed. Nine meetings were observed. Four SU records were reviewed. Analysis considered the extent to which change was created (section 4.1). CMOCs were used to create a refined programme theory (PT). The refined PT is summarised diagrammatically in Figure 3, including the extent to which it aligns with the IPT. The refined PT is explored in more detail below: section 4.2 describes refinements to the impact of change on SUs; a refined understanding of the mechanisms and contextual factors causing change is explored in section 4.3. Context-mechanism-outcome configurations can be found in appendix 2 and are cross-referenced within the text in section 4.3.

### 4.1 Extent of cultural change

Analysis demonstrated good adoption of the expected cultural changes; most staff embraced new ways of thinking and doing. Staff understood integrated working to benefit SUs, and were working together across and between voluntary and statutory organisations.

“‘Please don’t keep asking me to repeat the same stuff which is traumatic and retraumatizing. I’ve already told 15 of your colleagues this over my 20-year history of being involved in mental health services. Stop asking, you’ve got it all’, and I thought it made brilliant sense.”

“If a client rings me up and says I’m mentally ill, I need some help with, my mental health I don’t say to them. ‘Oh, you’ve come through to [VCSE provider], you need [other VCSE provider].’ I’ll take their details and I’ll talk to them about a bit, and I’ll see if they’ve got any advice needs and I will then say to them, ‘you know, okay, so what I can do for you is I can ask my colleagues in [other VCSE provider], to give you a ring, would you like that?’”

Where cultural change was slower this was understood as a work in progress, that change was still permeating some parts of the system, but that there was an inevitability of full integration. Causes of variation in cultural change are discussed in 4.3.

“It’s almost like lava. You know, how, how you see lava moving down a volcano and it doesn’t go enormously fast, but it kind of creeps along, filling, going in through gaps, and just filling the spot, and, and well, you know, it meets somewhere and then it kind of clags up a bit, and then has to find another way round.”

### 4.2 Impact of changes on service provision

As anticipated by the IPT, cultural change resulted in improved SU outcomes. A refinement of the IPT is an expansion upon what ‘improved SU outcomes’ means. These can be understood as distal outcomes within the refined PT; they are described in more detail below.

Everyone within the dataset who asked for help was offered a service, either from a voluntary or statutory provider, or both. This is a large departure from previous provision.

“There’s no gap now. There was a massive gap before, so people had to be really poorly to get a community mental health intervention.”

It was easier for SUs to access services: SUs were now able to access services via multiple routes, without the need for new primary care referrals. Routes included: GP, Home Treatment Team, Police, Primary Care Liaison Staff, Voluntary Sector, the Hospital. There was early evidence of people who had previously received an OMH service self-referring directly into the new system if new needs arose.

“People come into services in all different ways.”

“They [SUs] email straight into [our] inbox sometimes.”

Analysis showed SUs routinely being offered services based on their needs, rather than arbitrary thresholds of care based on their diagnosis. This included a wider range of services being offered to people, people being offered support who had previously been denied help, and offering integrated services.

“One of my colleagues came across a client sat outside our offices. Sat on the ground, just crying and threatening to strip all his clothes off so he could, could get arrested because his benefits had stopped, and he had no money. […] so she bought him to us, and we took him to Mind who deescalated his crisis, you know, calmed him down, did the therapeutic bit and then we contacted the DWP [Department of Work and Pensions] and sorted his benefits out. And then somebody from Second Step, sorted out a food parcel and accompanied him home […] And that was just really good, and that was. So, that’s not any traditional referral route. That’s just we found this person in the street. Erm, and it was sorted.”

A case study example of an integrated offer:

- Primary Care Liaison worker, Psychiatrist, Home Treatment Team, Talking Therapies and Second Step worked together care to support a person with long history of anger, trauma, self-isolation, voice hearing and intrusive thoughts. This person was offered a medication review, high intensity CBT, and a VCSE support worker.

### 4.3 Causes of change

This section discusses both mechanisms of change and contextual barriers and enablers that made integration harder or easier to implement.

#### 4.3.1 Language as a mechanism of change

As predicted by the IPT, staff understood the use of new language to have contributed to the high levels of integration in the early stages of implementation (CMOC 1). However, in a refinement to the IPT, staff currently utilised the new language to prompt one another if people were observed defaulting to old ways of working: language also functioned to sustain changes (CMOCs 2 and 3).

“Because we’re so, so embedded within Open Mental Health; I don’t think it [no wrong door]…it wouldn’t necessarily come up in conversation. I think people will, if something has happened that has not expressed thing of a…you know, no wrong door, they’ll say, ‘this is supposed to be a no wrong door service’.”

Notably, it was not necessary for staff to approve of the language used to adopt the underlying ethos and new ways of working. For example, many undertook ‘warm transfers’ by adapting the new language to their tastes. Instead, these staff referred to “friendly transfers” or “transfers”. This was sufficiently distinct from the old language of “referrals” to still prompt changes in thinking and working (CMOC 4).

The timing of the evaluation meant that analysis demonstrated language making smaller contributions to cultural change and integrated working than predicted by the IPT. Other casual factors unpredicted by the IPT were identified: relationships and communication; statutory staff understanding of the value of voluntary sector services; flexible and adaptive working. These are explored below.

#### 4.3.2 Relationships and communication

Improved communications and relationships were key resources in creating integrated working. For example, improved relationships enabled staff to have informal conversations, preventing reassessment when working with other services (CMOC 5).

“You get the odd individual challenge with the odd client, where you think they’re suitable for a service, and a service doesn’t [agree], but I think those are very few and far between. […] because we have such good relationships now with all these services.”

##### 4.3.2.1 Meetings as a strategy for relationship building

Improved communications and relationships were primarily created in online video meetings. Observations showed meetings being used to undertake joint decision making regarding the right care at the right time, but also to form professional relationships that enabled new ways of working to happen outside of the meetings.

“I think we’ve kind of established good working relationships by kind of having regular meetings with all the partners involved and kind of that sort of communication. And I think that’s key to it, whether you’re physically co-located or not […] having those open lines of communication.”

“Once you’ve seen somebody on the screen, it’s much easier then to go send an email saying, ‘Hi. I was really interested in what you had to say at the meeting, didn’t have a change to ask you, can we catch up?’, type of thing, you know, you’re…you’re then talking to a face, rather than just a name on an email.”

Once COVID-19 restrictions were lifted, away days, cross-training, and shadowing were also useful in improving relationships and communications. Analysis of this contextual factor suggests that online meetings are a strategy for creating relationship building, rather than a required resource in creating change: the required resource being the relationships created.

“We [IAPT] have, you know, had Open Mental Health [primary care liaison] come to our team meetings and talked about it [OMH], and what have you.”

##### 4.3.2.2 Changes in reasoning caused by improved relationships

Improved communication and relationships worked by creating the following changes in staff reasoning: 1. Understanding and embracing the new ways of working; 2. Understanding offerings provided by other services; 3. Feeling part of the same team; 4. Trusting other practitioners. These combined changes in reasoning meant that trusted assessments, warm transfers, and no wrong door were more likely to occur. This was because staff were less likely to suggest a transfer that might be inappropriate, and staff were more likely to accept a transfer as their personal relationship with the person increased their trust in that person’s assessment. Offers to SUs which involved joint working were more likely. Voluntary sector staff felt able to make warm transfers to other voluntary sector services, and statutory sector staff felt able to make warm transfers to both voluntary and statutory services.

“People, you know, are mutually respective [sic: respectful of?] organisations working together…seeing each other as part of a bigger team. You know, they’re all colleagues, as opposed to ‘us and them’, so yeah. That’s all part, that’s why I think it’s all working.”

“A shared understanding of each other’s services, and, and, what we do or don’t, don’t do.”

##### 4.3.2.3 COVID-19 as contextual factor affecting relationship building

COVID-19 restrictions were an important contextual factor affecting the relationship building mechanism. Prior to these restrictions, usual statutory ways of working meant that meetings were held in person. In this rural area, this would have restricted the frequency and attendance of all staff at meetings. However, COVID-19 meant meetings became remote by default, enabling more meetings with higher attendance across organisations, thus creating better relationships (CMOC 6).

However, remote working also made it harder to fully understand the other services provided within both statutory and voluntary sectors, making it difficult to know which services might contribute to providing the right service at the right time (CMOC 7). This contextual barrier was time-bound, lessening as people began blended working in the office enabling face-to-face meetings, and as team away days became possible.

“We were all literally working from home. So, people like the CMHT, Home Treatment, things like that, didn’t, didn’t see us visibly. So, I think, they were just like, ‘who are these people and what do they do?’”

“We’ve become more integrated, we used to have a separate office here, well, we often all would work remote. Now we will often… if I’m in the base, I will sit in the main office and have those conversations, and… and I feel very much now like we’re becoming that one team sort of approach.”

Interviewees also noted that COVID-19 had created a sense of urgency that prompted faster cultural change, as services pulled together in anticipation of increased mental health need. This enhanced staff from diverse organisations feeling like part of one team (CMOC 8).

“The reason Open Mental Health is up and running is because we had the COVID pandemic. I believe that if we hadn’t had the COVID pandemic we would have something up and running, but we’d still be talking about it and how to run it […] I think the predominant thing was is we thought we were gonna be a lot busier”

##### 4.3.2.4 Voluntary sector/statutory sector power imbalances as a contextual barrier to relationship building

An imbalance of power between statutory and voluntary sector providers, and voluntary sector perceptions of restricted statutory capacity and high thresholds of care provision worked as barriers to the relationship mechanism. Voluntary sector interviewees did not feel part of one team if meetings were run in a statutory style: utilising statutory language and focusing on the allocation of referrals into the statutory sector. Voluntary sector understandings of statutory capacity issues meant they felt disempowered to make warm transfers to the statutory sector (CMOC 9).

“It’s definitely moved from being a, I don’t know what you’d call it, a transactional sort of relationship to a partnership, but it is not an equal partnership. The NHS is the, very much the senior partner. Not in a derogatory way or a, and I’m not saying that in, in, but there is a sense of we easily, so culturally we easily move clients between Mind, Citizens Advice, Second Step. It’s always a bit of a thing to move a client back into the NHS. So, the difference would be is, say [VCSE staff member], who works for me, she will easily ring up Mind and sort something out. If she wants to get somebody seen by the NHS, she’ll ring me and she’ll just say, oh [VCSE manager], I’ve got this client this, this, this, this and this and should I refer them to the NHS? Yeah. Well how shall we do that or who should we contact? You know it’s always a bit more. You know? […] Yeah, a bit more of a thing.”

“I think Open Mental Health [primary care liaison] are really good at thinking of the Voluntary Sector as a way of supporting our clients, [and] I think the Care Coordination side is catching up with that…but…I feel we don’t get referrals from the Voluntary Sector to our [Trust based] service very much.”

##### 4.3.2.5 Pre-existing staff values as a contextual factor

A final contextual factor impacting on the relationship building mechanism was existing staff values. Where staff values already aligned with integration this acted as a contextual enabler, helping staff understand and embrace new ways of working (CMOC 10). All interviewees aligned with the changes, expressing the belief that changes benefitted SUs.

“[previous ways of working meant that] you’re having to be really rigid with thresholds…and that was really sad. This is people in our communities that are just gonna get more unwell, until they’ll need it. That’s not the way of working with people, is it? That’s not why we come into these professions.”

For some interviewees, particularly in the voluntary sector, these values were due to personal or professional experience of MH services.

“He [my friend] was discharged and then he went off and took his own life.”

“I worked in the voluntary sector before, and it was quite frustrating trying to get people to get support from the CMHT.”

#### 4.3.3 Statutory staff understanding of the value of voluntary sector services

Integration was partially driven by statutory staff understandings of what constituted appropriate and effective support for people with mental health problems. Statutory staff perceived that people with MH problems could be supported by addressing immediate needs that were often psychosocial in nature, and could not be addressed by statutory service offerings. Old ways of working were perceived as lacking purpose, reducing people’s agency, contributing to long waiting lists, and overly risk adverse. Integrated working across and within the voluntary sector, and primary and secondary care was understood to both fill treatment gaps and improve the quality of services provided. This motivated statutory staff towards integrated provision (CMOC 14). It was unclear to what extent this shift towards psychosocial, recovery-orientated, understanding already existing within staff (i.e., a contextual factor), or was prompted by mechanisms such as language (e.g., ‘right care at the right time’) (CMOC 11) and the introduction of a needs-based assessment tool (CMOC 12). However, it was clear that this required reasoning regarding the value of VCSE services was distinct from the contextual disabler of pre-existing VCSE-statutory power imbalances discussed in section 4.3.2.4.

“We’ve moved far, far away from this working with people *ad infinitum*. You know, going around for a cup of tea once a week for 12–20 years […] the case is actually that, ‘At this time you have these needs, we’ll offer an intervention based around that.’”

“It’s very intervention focused: we’re going to help you progress we’re going to help you recover, and you’re going to move up and down this pyramid [of different services].”

Additionally, statutory staff perceived that working with VCSEs could fill statutory sector treatment gaps: both preventing MH problems from escalating, and providing support after statutory service support is complete, preventing relapse. This reasoning helped statutory staff embrace integrated working. Again, it was unclear whether this perception was pre-existing or caused by changes in working culture (CMOC 13).

“If the people feel like they’ve left [been discharged to nothing] and they’ve got no support and network they’re generally going to…they’re going to bounce like a bouncy ball. They’ll hit the ground and they’ll go…escalate right up to the top again, and then nothing…nothing’s changed for that person. Whereas, if you can […] get them a support network that’s not a [statutory service] professional, you’re probably going to get better results […] if you can create a network around somebody that’s helpful and healthy, it’s probable that they’re not going to bounce up quite so high next time.”

Contextual barriers to creating this change within statutory staff existed: some parts of statutory services had rigid ways of working that were set by national policy and/or strict diagnostic-based ‘pathways’ of care. People working within these services found it harder to adopt integrated working (CMOC 15). Notably, these services were often disempowered from using the DIALOG+ tool, further suggesting the tool acts as a resource in adopting a psychosocial, recovery-orientated understanding that aids integration with the voluntary sector (CMOC 11). These services included the nationally-dictated Improving Access to Psychological Therapies [IAPT] service.

“The assessment information that we get from a DIALOG[+] is not sufficient to, for us to be able to really target the most appropriate intervention. So, [IAPT] would always have to offer another assessment which flies in the face of actually what we’re trying to achieve […] And that’s the roadblockishness of IAPT.”

“For PTSD [post-traumatic stress disorder], for example, […] it would always have a high intensity, either cognitive behaviour, trauma focused cognitive behaviour therapy or EMDR, erm, intervention. So, that’s a very specific model and in order to identify whether this person is presenting with PTSD or not, we need to elicit, you know that the, the very specific info, into, information around trauma symptoms that allows us to identify that.”

#### 4.3.4 Flexible and adaptive working

Staff and managers in the voluntary and statutory services reported that being able to, and feeling comfortable to, work flexibly was an important mechanism for changing to integrated working (CMOC 16). This included job roles that were iteratively defined, working flexibly in budget management, employing staff, governance, team structures and organisation of services.

“I’m an Occupational Therapist and they hired me [as Primary Care Liaison lead] by accident because they actually wanted a non-[medical] prescribing nurse [NP], but I turned up for the interview and I was like, ‘I’m not that, I’m an OT,’ and they had to continue with the interview and they sort of formulated a role for me, which was amazing, and so that’s why they hired [Non-Medical Prescribing Nurse] as well to work alongside to fulfil the NP part of the role, so we’re quite lucky in the Mendips in that there are two of us, and I think that’s given us greater flexibility in terms of thinking about service development and also being more creative together because we’re able to bounce ideas around a little bit more.”

“I would say sort of 60 to 75% of the transfers into [particular VCSE provider] come through me [manager] and the rest then do go in through all sorts of routes and I get told about them. But, I’m happy with that ‘cause I quite like floppy systems, erm, I tend to think they work better for clients.”

Adaptive and responsive management was a key resource in improving integration. For example, mangers re-organised meetings, recruitment and line management to address power imbalances, and designed away days in response to feedback that it was difficult for staff to understand the full OMH offering. This included rotating meeting chairs between VCSE and statutory staff, less reliance on statutory service SU identifiers and language, cross-organisation supervision, and recruiting statutory staff who had experience of working closely or within VCSE organisations.

“Second Step invited me [Trust member of staff], so I started clinically supervising some of the Voluntary Sector partners […] When, when this [VCSE staff member] left her position, [VCSE staff] asked if I would like to co-interview with her so it was me and [VCSE staff] interviewing together, which was lovely and that was proper like joint working.”

Trust acted as a contextual enabler to flexible and adaptative working by team leaders, managers, and staff (CMOC 17). This included: trust from strategic leaders; trust from team managers; trust from the frontline staff. Trust between managers at different levels involved having justification for new ways of working, especially if these were novel or unusual. Trust from frontline staff involved belief that the manager would support them with any challenges/difficulties whilst using the new ways of working, this was contingent on staff having previous experience that the manager “had their back”. This highlighted an advantage of a strategic level, top-down cultural approach.

“I think if you have an open culture that’s quite accepting of change, and the team is managed that way, I’m going to say from the top, actually, and I don’t like that word, but from management, I think the individuals within it are more keen to give change a go.”

Contextual barriers to flexible and adaptive working also existed. People who had started a new role during the COVID-19 restrictions were less likely to feel safe in undefined roles, lack of face-to-face feedback reduced their confidence that they were performing their role as expected (CMOC 18). People employed in the more rigidly-defined statutory services were disempowered from flexible working by the nature of the service, for example if the nature of service provision was dictated at a national level (CMOC 19).

“Very bad for me [starting this job during COVID lockdown]. I would have weeks when I would finish my hours and think what have I done this week? Have I earned my money this week, type of thing, which I think is made more difficult when you’re working at home, nobody knows what you’re doing?”

“It feels as though IAPT is quite angular, and it sits within something that is trying to be quite fluid.”

If staff had a varied employment history, with a range of transferrable skills, this acted as a contextual enabler to flexible and adaptive working. These staff felt more comfortable with the flexible and adaptive ways of working that empowered integrated working (CMOC 20).

“With skills in, in all sorts of areas. Whether that’s being a Nurse, a Teacher, erm, working in the Voluntary Sector, and I’d say, yeah, just lots of life skills that they’ve, they’ve kind of built up along the way […] I’ve worked in the Voluntary Sector. I’ve worked in communication and campaigns role, I’ve yeah, I’ve kind of worked in Private, Public and Voluntary Sector.”

## 5. Discussion

This evaluation aimed to investigate the extent of integration between and within voluntary and statutory sector MH services, the impact of integration on service provision, and how, why and under what circumstances integration took place. The evaluation found some evidence that a cultural change approach, where strategic leaders created changes to language, thinking and ways of working, led to integrated working. Service level changes included a reduction in repeat assessments, increased routes into services, joint working, reduced cliff-edges of care and improved transfers between services. This integrated working created large-scale changes in the services offered: all SUs were offered support, and more joined-up offerings were used. This adds to existing evidence that integrated care can help address treatment gaps [27]. Mechanisms of change included: relationships and communication, statutory staff understanding the value of voluntary sector services, flexible working. Relationships and communication were enabled by reducing imbalances of power between providers. Where statutory services were rigid in provision this made valuing voluntary sector services more challenging. Trusting and supportive management enabled flexible working.

This study expands what is known about the role of alliance-style governance arrangements (i.e., where each organisation retains autonomy, but work together as a coalition) in integrating mental health services [28]: A shared vision/goal consensus in the form of what integrated working practices should look like (via a cultural change model) empowered integrated working between and within statutory and voluntary sector services. As Wiktorowicz et al. note [28], this required trust between alliance members. This study shows this trust involves people from different organisations building relationships that allow them to feel part of the same team, understand the role of different services, and trust other practitioners from different services. Trust also involves staff at different hierarchical levels trusting one another: feeling safe as they implement the flexible and adaptive working practices required for integration of both governance and service delivery.

Additionally, this study adds to existing evidence regarding how permission to operate flexibly, (i.e., without rigid rules, ways of working and strict oversight), enables managers and staff to implement changes in how health care is delivered. Existing evidence suggests that strategic level leadership and support, with managers permitted to develop detail regarding how implementation takes place, leads to improved adoption of new health care working practices [29]. The findings support this evidence: strategic leaders designed and supported cultural change towards integrated working, and allowing flexible implementation by managers acted as an enhancer of change. A novel addition is that permission for frontline staff to be flexible in how they worked with SUs was also required. The realist approach highlighted contextual factors affecting ability to work flexibly not reflected in existing evidence: two-way trust between different hierarchies of staff enabled flexible working; remote working disables ability to build trust; varied career pathways that included experience of different ways of working acted as an enabler. Finally, the realist focus on causation highlighted how permission for managers to work flexibly enhances implementation: allowing managers to address factors that may not have been accounted for by strategic leaders, including power imbalances between organisations.

A novel finding is the requirement for frontline staff and managers to understand the value of integrated working. This includes the understanding that integrated working improves service provision by preventing denials of care, and the understanding that mental health problems can be prevented and treated via psychosocial interventions that are often best provided by or with the voluntary sector. It is likely that the use of a needs-based assessment tool [30] may enable this latter understanding within statutory service staff. Importantly, system-level contexts act as barriers here: rigid diagnostic pathways disable the use of the needs-based assessment, and rigid national policies regarding statutory service provision act as barriers to statutory staff valuing integrated working.

### 5.1 Strengths and limitations

A strength of this study is the use of ethnographic and internal document data via the RiR approach within a realist evaluation. By embedding RiRs at both strategic and local team level it was possible to create a detailed and rich understanding of the contextual factors affecting change. This detailed understanding was enabled by focusing on one locality, yet the use of realist understandings of context has allowed for the creation of generalisable knowledge from this small sample frame. However, it should be acknowledged that these findings are limited by the small sample frame, particularly of SU records, and difficulty in interviewing SUs. This has resulted in the impact of SU contextual factors being underexplored in this work.

## 6. Lessons learned

- Ecosystems of care looking to address MH treatment gaps should consider statutory-voluntary sector integration
- Cultural change (via changes in language and ways of working) is a useful tool in implementing and sustaining the integrated statutory-voluntary sector provision
- Cultural change is insufficient for implementing integrated statutory-voluntary sector working. Implementation also requires: improved communication between staff in different services; a psychosocial recovery orientated understanding of mental health problems by statutory staff; flexible and adaptive working at all levels; strategies for addressing power imbalances
- Implementers of integrated statutory-voluntary sector working must be mindful of, and have strategies to address, the rigid diagnostic pathways and national policies within statutory services that can act as a barrier to integration

## 7. Conclusion

Integration between and within statutory and voluntary sector MH service providers, using a culture change model, addresses gaps in MH care. Service level changes included a reduction in repeat assessments, increased routes into services, joint working, reduced cliff-edges of care, and improved transfers between services. Mechanisms of change included: relationships and communication, statutory staff understanding the value of voluntary sector services, flexible working. Relationships and communication were enabled by reducing imbalances of power between providers. Where statutory services were rigid in provision this made valuing voluntary sector services more challenging. Trusting and supportive management enabled flexible working. Optimal implementation requires addressing contextual barriers.

## Additional File

The additional file for this article can be found as follows:

10.5334/ijic.9060.s1 Appendices. Appendix 1 to 2.
