# Supplementary material for: What Works, for Whom, in What Circumstances and Why, When Integrating Voluntary and Statutory Community Mental Health Services: A Realist Evaluation Case Study
Source: Int J Integr Care. 2025 Sep 30;25(3):31. doi: 10.5334/ijic.9060 (PMC12493028; doi:10.5334/ijic.9060)
Supplement: Appendices. — Appendix 1 to 2. [file ijic-25-3-9060-s1.pdf]

Appendix 1: voluntary sector provision within Open Mental Health

Reproduced with permission of from *Rethink Mental Illness* from https://openmentalhealth.org.uk/directory-of-partner-services/. Voluntary sector provision fluctuates, and this list should be seen as indicative; it was accurate as of 17/04/2025. Due to the scope of the Framework, it excludes any provision for people under 18. It excludes any provision by organisations that have joined the alliance post-evaluation.

**Name of service**<br>*Service provider(s)*	**Service description**
Mindline Somerset 24/7 Helpline<br>*Mind in Somerset*	Mindline Somerset's helpline offers emotional and mental health support 24 hours a day, seven days a week. As a confidential listening service, Mindline provides a safe place to talk and information on local services for Somerset residents. It's one of the main access points for Open Mental Health services.
Community Rehabilitation<br>*Second Step, Mind in Somerset, Rethink Mental Illness*	The Open Mental Health Rehabilitation Team supports people with complex mental health difficulties to achieve their goals and gain skills and confidence to live as independently as possible. The service was set up in April 2023 as part of NHS Somerset's transformation of mental health services across the county. People are supported for the most part in the community as we create a system which avoids hospital admission. The Rehabilitation Team is linked with key services across the county, including Open Mental Health, the Recovery College, and NHS inpatient and community teams. There are also strong links to partner organisations outside the NHS, including housing and voluntary sector services. From its inception, the Rehabilitation Team has been designed and shaped with the support of people with direct lived experience of coping with long-term mental health difficulties. Referrals and transfers to the service come from Somerset Foundation Trust's teams only for the time being. Referrals will be made electronically from a person's current care team.

Crisis Safe Space<br>*Mind in Somerset, Second Step, Watch*	Crisis Safe Space offers a community crisis alternative in each locality tailored to the needs of people in those areas. The key objective is to create safe and welcoming spaces in the community that people can access in a crisis out-of-hours. Appointments can be made face-to-face or virtually and can be booked through the Open Mental Health website or through the Mindline Somerset helpline.
Eating Disorders<br>*SWEDA*	Fully trained and supervised by SWEDA, clinical leads offer 1-2-1 support in this specialist area of mental health. Knowledgeable and qualified workers have a thorough understanding of this illness, the specialist treatment it requires, eating disorder treatment structures available and a comprehensive brief working across all levels in the county.
Family Safeguarding<br>*Balsam Centre, Mind in Somerset, Second Step*	As part of the Open Mental Health model, adult Mental Health Practitioners (Recovery and Wellbeing Workers) are employed by Mind, Second Step and the Balsam Centre and are embedded within children's social work teams. Our practitioners are also closely linked with the Open Mental Health locality teams and the wider Open Mental Health model. Family Safeguarding teams use a multi-disciplinary approach to provide high-quality support to families with complex needs. By working with adults with mental health issues to achieve their goals, we enable families to remain together and improve outcomes. A VCSE employed Clinical Psychologist oversees the clinical supervision of staff within the Family Safeguarding team, providing case management, oversight and group supervision.
Group Activity Specialism<br>*Mind in Somerset*	Group-based therapeutic activities and interventions can support the recovery journeys of people with mental health challenges and are a valuable component of holistic care. Amongst other things, group activities include shared reading, arts and crafts, self-care-focused peer support, cooking,

	getting online in a Zoom, and a recovery and wellbeing group. The goal is to create a safe and supportive group environment where people can learn, grow and work towards improving their mental wellbeing. Online sessions are available to anyone over 18 to explore ways to stay well. Each session includes information on specific topics, time for discussion relating to the topics covered and, more generally, an opportunity to meet and speak with others in the community. We also offer support groups and hearing voices peer support in various localities.
Health Education England Peer Support Worker Training<br>*Watch*	Peer support is a style of helping where individuals who have experienced mental health problems come together for mutual support and learning through sharing techniques and strategies for maintaining their wellbeing. Building relationships with peers can connect people with others who know and understand.
Older People's Support<br>*Age UK*	Friendly Phone Calls is a free service for those experiencing the debilitating effects of chronic loneliness. Suitably trained volunteers visit clients in their own homes. They listen, laugh, share stories and memories and, through guided conversation, begin discovering what would make a difference. Ultimately, the aim over time is to help a lonely older person become more involved in their community.
Peer Connections<br>*Rethink Mental Illness*	Peer Connections is a free service offering support to Somerset residents aged 18+ who experience challenges with their mental health. Our peer volunteer team have had their own challenges with mental health and discovered practical coping strategies and skills. A peer mentor will listen without judgment, offer support based on their lived experience, allow service users to think about what they'd like to achieve and share ideas that have worked for them.

Peer Support<br>*Watch*	The ethos of Chard Watch is to support individual peers and peer groups with a person-centred approach and not to “fix it” for group members. We offer solid support for members of peer groups and their peers through the provision of timely interaction and information. We deliver a comprehensive support package for existing and new peer groups county-wide via support workshops that address some common issues peer groups face.
SMI Physical Healthchecks<br>*Rethink Mental Illness*	Annual Physical Health for People with Serious Mental Illness or SMI – usually includes anyone diagnosed with schizophrenia, bipolar disorder or other psychosis and other mental health conditions. Checks are designed to help people living with a severe mental illness and their clinicians to pick up early signs of physical health issues such as diabetes, stroke or heart problems in order to take action before these issues become more serious.
Step Down<br>*Second Step, Watch, and Somerset County Council*	The Step Down service supports people back into community life after spending time in psychiatric inpatient care. We offer a trauma-informed approach to all the support we give, ensuring every client is treated as an individual with their own unique experiences and needs. We help with budgeting and make onward referrals to specialist support if needed. We also help with managing benefits. For example, we will help with applications for universal credit and personal independent payments (PIP). Alongside this practical help, we offer emotional support helping to build people’s confidence and resilience to reconnect back into their communities. Supporting clients to make strong community links is a vital part of their recovery and helps people to avoid going back into hospital.
Step Up<br>*Rethink Mental Illness, Watch, Mind in Somerset*	The Step Up Service is a CQC-registered Crisis House based in Yeovil, Somerset, and open to county-wide referrals from the local NHS Home

	Treatment Service. The Crisis House provides short-term accommodation for up to six people experiencing a period of crisis with their mental health.
Step Ladder<br>*Second Step*	Stepladder creates safe spaces for men in Somerset, offering a place to talk, get involved in new groups and activities, and connect with the community. It is not set up to deliver services directly but works alongside local organisations to develop relationships, build trust, and raise awareness about men's mental health. It plays an important role in early suicide prevention. The project also offers grants of up to £1000 to help fund groups, projects and initiatives for men in Somerset.
Suicide Prevention Peer Support Workers<br>*Mind in Somerset, Second Step*	Our Peer Suicide Prevention Project forms part of a wider suicide prevention strategy for Somerset and is funded through the Open Mental Health alliance. As Peer Support workers, we use our own lived experience to work with people face-to-face. Our work in Somerset is person-centred and can include formulating safety plans for those in deep distress, Wellbeing Recovery Action Plans (WRAPs), and the Five Ways to Wellbeing as part of our ethos for continued wellbeing and recovery.
Time to Connect<br>*Mind in Somerset*	Time to Connect Somerset is a campaign to challenge stigma and discrimination around mental health. The work at the Time to Connect Somerset hub is driven by Time to Connect Champions. TTC Champions are a supportive network of people with lived experience of mental health issues who recognise that negative stereotypes about mental health can sometimes hold us back, preventing us from leading our best lives.
Stakeholder Engagement<br>*Spark, Diversity Voice*	An OMH Stakeholder Engagement Coordinator works alongside OMH Champions and Diversity Voice to increase engagement with the wider VCFSE sector and build upon some of

	the recommendations in the Community Engagement end-of-project report.
Hearing Voices Group Activity<br>*Mind in Somerset*	Hearing Voices groups provide safe places for people to talk about their experiences, which can be distressing and overwhelming. Some people feel that their voices are positive and supportive but still need an outlet to share them with others. Mind in Somerset active listeners facilitate the meetings and ensure attendees feel safe, accepted and able to share their experiences with other voice hearers.
Suicide Prevention Outreach<br>*SASP, Somerset County Council Public Health*	Somerset Activity and Sports Partnership (SASP) engages community members through physical activity. The goal is to build relationships through fun and casual sports like badminton, ping pong or walking. The program supports different groups, including younger adults, men, veterans, etc. Qualified staff encourage conversations about mindset, energy levels, family and social connections, and more. This helps increase self-esteem, self-worth, social connectedness and confidence. One-to-one peer mentoring is provided where possible. Individuals can connect between sessions via phone, e-mail, or video link to discuss their wellbeing journey.
Wider Determinants<br>*Citizen's Advice*	Citizens Advice offers specialist casework and advice to clients across the whole spectrum of advice needs, including face-to-face, ward-based, and virtual support, but with a particular focus on debt, money advice, welfare benefits, and housing.

Appendix 2 : CMO Configurations

**Use of language to create cultural change which prompts integrated working**<br>*Section 4.3.1*
1. If the new language is adopted by staff (mechanism: resource), then this will

prompt adoption of new ways of thinking (mechanism: reasoning) and working (outcome).
2. If the early extent of change is good and staff turnover is low (context), then the adoption of the new language (mechanism: resource) quickly ceases to impact the adoption of new ways of thinking (mechanism: reasoning) and working (outcome).
3. If the extent of change is good (context) then the new language (mechanism: resource) can be used as prompts to improve maintenance of new ways of thinking (mechanism: reasoning), improving sustainability of change (outcome).
4. If the new language is not acceptable to staff (context), then staff will adopt similar language (mechanism: resource), then this language will prompt the adoption and retention of new ways of thinking (mechanism: reasoning), and working (outcome)
**Use of good communications and relationships to empower integrated working**<br>*Section 4.3.2.*
5. If staff members from different organisations have good communications and relationships via meetings, away days, cross-training, and shadowing (mechanism: resource) then they will 1. Understand and embrace the new ways of working, 2. Understand offerings provided by other services, 3. Feel a part of the same team, 4. Trust other practitioners (mechanism: reasoning) making them more likely to work in an integrated way (outcome).
6. If the working environment is conducive to online working (context) then this increases the frequency with which staff can meet to improve communications and relationships (mechanism: resource), making it more likely that they will 1. Understand and embrace the new ways of working, 2. Understand offerings provided by other services, 3. Feel a part of the same team, 4. Trust other practitioners (mechanism: reasoning), making them more likely to work in an integrated way (outcome).
7. If remote working is the default (context) then when staff meet to improve communications and relationships (mechanism: resource) this limits the improved understanding of the offerings provided by other services (mechanism: reasoning), reducing the adoption of integrated working (outcome)
8. If there is an external factor (e.g., COVID emergency) that creates a sense of urgency (context) then when staff meet to improve communications and relationships (mechanism: resource), this is more likely to lead to people feeling a part of the same team (mechanism: reasoning), making integration more likely (outcome).
9. If there is an imbalance of power between partners (context) then when staff meet to improve communications and relationships (mechanism: resource) this is less likely to lead to people feeling like part of the same team (mechanism: reasoning), making integration less likely (outcome).
10. If the existing values of the individual staff members match the ethos of integration (context) then when staff meet to improve communications and relationships (mechanism: resource) this is more likely to lead to people feeling a part of the same team (mechanism: reasoning) and more likely lead to staff understanding and embracing the new ways of working (mechanism: reasoning), making integration more likely (outcome).

**Focus on purposeful, psychosocial, recovery-orientated interventions highlights the values of integrated working**<br>*Section 4.3.3.*
11. TENATIVE: If staff adopt a needs-based assessment tool (mechanism: resource) then this will lead to statutory staff understanding that purposeful, psychosocial, recovery-orientated interventions benefit SUs (mechanism: reasoning) then this makes them more likely to embrace working with the voluntary sector (outcome)
12. TENATIVE: If staff adopt the 'right care at the right time' language (mechanism: resource) then this will lead to statutory staff understanding that purposeful, psychosocial, recovery-orientated interventions benefit SUs (mechanism: reasoning) then this makes them more likely to embrace working with the voluntary sector (outcome).
13. TENATIVE: If staff already have a psychosocial, recovery-orientated, understanding of how best to address mental health problems (context) and (mechanism: unknown resource/s) then this makes them more likely to embrace working with the voluntary sector (mechanism: reasoning) leading to better integration (outcome).
14. TENTATIVE: If statutory sector staff understand that voluntary sector services can contribute to a psychosocial service provision that acts both to prevent and treat mental health problems (context) and (mechanism: unknown resource/s) then this makes them more likely to embrace working with the voluntary sector (mechanism: reasoning) leading to better integration (outcome).
15. If statutory services have rigid ways of working or strongly-defined diagnostic pathways (context) and if staff adopt a needs-based assessment tool (mechanism: resource) then the rigid ways of working will inhibit the tool leading to statutory staff understanding that purposeful, psychosocial, recovery-orientated interventions benefit SUs (mechanism: reasoning) then this makes them more likely to embrace working with the voluntary sector (outcome).
**Flexible and adaptive working makes it easier to make the changes necessary to implement integrated working**<br>*Section 4.3.4*
16. If staff and managers are able to (mechanism: resource) and feel safe to (mechanism: reasoning) work in flexible and adaptive ways then they are more able to make the changes to ways of working required to adopt integrated working (outcome).
17. If there is trust between strategic leaders, managers and staff (context) and strategic leaders are involved in defining and leading integration (context) then staff and managers are more able (mechanism: resource) and feel safer (mechanism: reasoning) to work in the flexible and adaptive ways of working that impower integrated working (outcome).
18. If there is an external factor (eg., COVID restrictions) that make face-to-face feedback difficult (context) and staff are permitted to work in a flexible and adaptive way (mechanism: resource) they are less likely to feel safe working in a flexible and adaptive way (mechanism: reasoning) making them less likely to adopt the flexible and adaptive ways of working that impower integrated working (outcome).
19. If staff work for a statutory service with rigid working patterns (context) this

	will disempower staff from working in a flexible and adaptive way (mechanism: resource) they are less likely to feel safe working in a flexible and adaptive way (mechanism: reasoning) making them less likely to adopt the flexible and adaptive ways of working that impower integrated working (outcome).
20.	If staff and managers have a varied employment history (context) and are permitted to work in flexible and adaptive ways (mechanism: resource) and they will feel safe to work in flexible and adaptive ways (mechanism: reasoning) making them more able to make the changes to ways of working required to adopt integrated working (outcome).
